# pH-Responsive Polyketone/5,10,15,20-Tetrakis-(Sulfonatophenyl)Porphyrin Supramolecular Submicron Colloidal Structures

**DOI:** 10.3390/polym12092017

**Published:** 2020-09-03

**Authors:** Esteban Araya-Hermosilla, Ignacio Moreno-Villoslada, Rodrigo Araya-Hermosilla, Mario E. Flores, Patrizio Raffa, Tarita Biver, Andrea Pucci, Francesco Picchioni, Virgilio Mattoli

**Affiliations:** 1 Center for Micro-BioRobotics, Istituto Italiano di Tecnologia Viale Rinaldo Piaggio 34, 56025 Pontedera (PI), Italy; 2 Laboratorio de Polímeros, Instituto de Ciencias Químicas, Facultad de Ciencias, Universidad Austral de Chile, Valdivia 5090000, Chile; mario.flores@uach.cl; 3 Programa Institucional de Fomento a la Investigación, Desarrollo e Innovación (PIDi), Universidad Tecnológica Metropolitana, Ignacio Valdivieso 2409, P.O. Box 8940577, San Joaquín, Santiago 8940000, Chile; rodrigo.araya@utem.cl; 4 Department of Chemical Product Engineering, ENTEG, University of Groningen, Nijenborgh 4, 9747AG Groningen, The Netherlands; p.raffa@rug.nl (P.R.); f.picchioni@rug.nl (F.P.); 5 Dipartimento di Farmacia, Università di Pisa, Via Bonanno 6, 56126 Pisa, Italy; tarita.biver@unipi.it; 6 Dipartimento di Chimica e Chimica Industriale, Università di Pisa, Via Moruzzi 13, 56124 Pisa, Italy; andrea.pucci@unipi.it

**Keywords:** polyketone, Paal–Knorr reaction, polymeric surfactant, TPPS, supramolecular nanostructures, pH responsiveness

## Abstract

In this work, we prepared color-changing colloids by using the electrostatic self-assembly approach. The supramolecular structures are composed of a pH-responsive polymeric surfactant and the water-soluble porphyrin 5,10,15,20-tetrakis-(sulfonatophenyl)porphyrin (TPPS). The pH-responsive surfactant polymer was achieved by the chemical modification of an alternating aliphatic polyketone (PK) via the Paal–Knorr reaction with N-(2-hydroxyethyl)ethylenediamine (HEDA). The resulting polymer/dye supramolecular systems form colloids at the submicron level displaying negative zeta potential at neutral and basic pH, and, at acidic pH, flocculation is observed. Remarkably, the colloids showed a gradual color change from green to pinky-red due to the protonation/deprotonation process of TPPS from pH 2 to pH 12, revealing different aggregation behavior.

## 1. Introduction

Supramolecular chemistry is an effective approach to obtain functional structures from building blocks linked together by noncovalent (and reversible) interactions [1,2,3,4,5]. Notably, functional building blocks work as individual constituents that synergistically contribute to the intrinsic properties and final functionalities of complex systems [6]. These systems can respond dynamically in the presence of external stimuli due to the transitory character of the noncovalent interactions [7]. The reversible character of supramolecular interactions between functional building blocks allows for the formation of highly complex organized matter with novel properties [8,9,10,11]. Supramolecular structures find applications in light-harvesting [12,13], pharmacology [14], regenerative medicine [3], and catalysis [15]. Among several approaches to fabricate supramolecular systems, electrostatic self-assembly is one of the most attractive due to its easiness, low cost, and versatility in terms of building block choices [5,6,15,16]. Two steps compose this method. First, the constituents approach each other and self-assemble by electrostatic interactions. Secondly, the system grows by cooperative binding mechanisms, such as π−π staking, hydrophobic/solvophobic interaction, and hydrogen bonding [17,18].

Polymeric surfactants [19,20,21,22] are remarkable macromolecules that can act as building blocks in supramolecular and colloidal systems [11]. Due to their lyophilic/lyophobic features, they have been used to prepare emulsions and suspensions [23,24] for drug delivery systems [25,26], in photodynamic therapy [27], and oil recovery [28,29,30], to give a few examples. For these applications, the precise control of molecular weight and the balance between hydrophilic and hydrophobic segments are critical parameters for a controlled performance [31]. To accomplish these conditions, amphiphilic polymers are synthesized by different chemical routes. Among them, living radical polymerization approaches such as atom transfer radical polymerization (ATRP) [32,33], reversible addition-fragmentation chain transfer polymerization (RAFT) [34,35], and nitroxide-mediated polymerization (NMP) [36] are widely used. It is well known that these polymerization methods are cumbersome and expensive [37]. An alternative is represented by the chemical modification of alternating aliphatic polyketones with primary amine compounds via the Paal–Knorr reaction [38,39,40,41,42,43,44]. Throughout this method, hydrophilic/hydrophobic functional groups are grafted randomly on the polyketone backbone as pendant groups, producing random amphiphilic polymers [38,45]. The Paal–Knorr reaction on polyketones shows appealing characteristics such as easiness and cheapness, solvent- and catalyst-free, quantitative yields, and water as the only by-product [46,47]. Besides, there are a myriad of commercially available primary amine compounds, allowing the chemical modification of polyketones with acid, basic, aromatic, and aliphatic groups [47]. The robustness opens the possibility of synthesizing smart polymers such as pH-responsive [38] and self-healing materials [39,40], emulsions with adhesive properties [46,48], and coatings [49].

**Figure 1:**
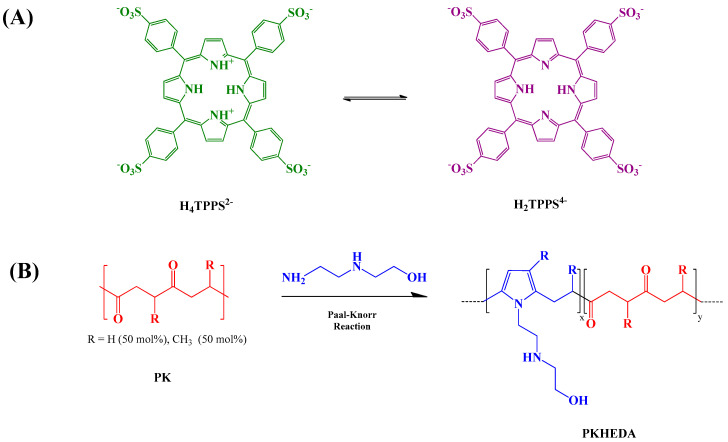
(**A**) Acid (left) and base (right) form of 5,10,15,20-tetrakis-(sulfonatophenyl)porphyrin (TPPS). (**B**) Schematic representation of the Paal–Knorr reaction on the polyketone (PK) with N-(2-hydroxyethyl)ethylenediamine (HEDA) [84].

Porphyrins are attractive molecules to design functional supramolecular structures [15,50]. Essentially, synthetic and natural porphyrins are composed of four pyrrole rings interconnected by four methine bridges. Two of the four pyrrole rings can accept protons at acidic conditions, or be used to coordinate the dye with metal ions [51,52]. The aggregation of porphyrins is of particular interest in various technological applications [53]. For instance, Schwab et al. designed a method to self-assembled porphyrin as nanorods that displayed photoconductive properties [54]. In another research work, Komagoe et al. synthesized porphyrin aggregates by interacting two opposite charged porphyrins to enhance the production of hydrogen peroxide under neutral pH [55]. The simple chemical modification of porphyrins benefits the synthesis of novel materials that are potentially applicable in areas such as photodynamic therapy [56,57], sonodynamic anticancer therapy [58], catalysis [59], sensors [60,61,62], and light-harvesting [63]. A particular water-soluble porphyrin, 5,10,15,20-tetrakis-(sulfonatophenyl)porphyrin (TPPS), is composed of a porphinato macrocycle meso-substituted by four hydrophilic sulfonate substituents (Figure 1a). When one dissolves this porphyrin in aqueous media, it may form two types of aggregates: J-aggregates and H-aggregates [64]. The formation of head-to-tail J-aggregates (see Figure S1) is initiated by the assembling of the TPPS di-anionic form (H_4_TPPS^2−^), since the negatively charged benzene sulfonate groups of one dye molecule place above the cationic center of the others; then, the ensemble arranges in tilted arrays. On the contrary, the tetra-ionic form of the dye (H_2_TPPS^4−^) originates the H-aggregates by forming a sandwich-like conformation (see Figure S1). Diverse factors control the aggregation of TPPS, such as pH [65], ionic charge [66], and dye concentration in the solution [67]. Additionally, the aggregation of TPPS may be achieved by its interaction with aromatic polymers [67], polyelectrolytes [68], dendrimers [69,70], micelles [71] and vesicles [72].

Charged porphyrins form supramolecular structures and colloids by interacting with oppositely charged polymers [15,73,74,75,76]. The polymer can induce the aggregation of porphyrins [77], as well as improve the stability of the aggregates in aqueous media [75]. The charged side groups of porphyrins undergo short-range electrostatic interactions with polymeric functional groups resulting in complex polymeric colloids [74,78]. One can control the structure and the optical properties of these systems by changing the pH and ionic strength of the media, as well as the dye/polymer concentration ratio [16,74,79]. The polymer/porphyrin assemblies may find use as pH-sensing systems caused by color change related to transitions between dispersed monomers, H-type aggregates, and J-type aggregates in the polymer domain by changing the pH, producing bathochromic or hypsochromic shifts readily followed by spectroscopic measurements and calibration [80,81,82,83].

In a previous work, we reported the formation of cationic complexes of low-concentrated TPPS and polymeric surfactants (PKHEDA) produced by the chemical modification of an alternating aliphatic polyketone (PK) with N-(2-hydroxyethyl)ethylenediamine (HEDA) via the Paal–Knorr reaction at different degrees of grafting (Figure 1b) [84]. Polymers with a lower degree of grafting resulted more hydrophobic. Novel submicron structures will be shown in this work consisting of the polymer/TPPS system using TPPS at high relative concentration and a PKHEDA surfactant polymer showing a high degree of grafted HEDA. The secondary amine and the hydroxyl groups of HEDA, along with the pyrrole groups, allow the polymer interacting with the negatively charged porphyrin TPPS by noncovalent interactions, hence supporting the formation of pH-responsive supramolecular negatively charged colloidal structures. We will study the influence of the pH on the polymer/dye system behavior by absorbance, size and zeta potential measurements, ultrafiltration experiments, and scanning electron microscopy.

## 2. Experimental

### 2.1. Materials

Aliphatic polyketones composed of ethylene, propylene, and carbon monoxide were synthesized according to a reported procedure [85], yielding a polyketone with an aliphatic part comprised by the 50 mol% ethylene and 50 mol% propylene (PK, Mw = 3640 g mol^−1^). The 2,5-hexanedione (Sigma-Aldrich, 98%), 5,10,15,20-tetrakis(4-sulfophenyl)porphyrin (TPPS, TCI 95%), and *N*-(2-hydroxyethyl)ethylenediamine (HEDA, Sigma-Aldrich 98%) were used as received. Distilled water and deuterated chloroform CDCl_3_ (Sigma-Aldrich) were used as solvents. Paal–Knorr reaction on PK with HEDA yielded the pH responsive PKHEDA (see Supplementary Section S1 for procedures). In addition, 2,5-hexanedione was also derivatized with HEDA to obtain a model low-molecular weight compound for characterization purposes (see Supplementary Section S2 for procedures). Sodium alginate (Büchi) and calcium chloride (CaCl_2_ × 2 H_2_O, Sigma-Aldrich) were used to prepare pH color sensitive hydrogels.

### 2.2. Characterization

The elemental composition of the functionalized polymer was determined by using an Elemental Vario Micro Cube (Milano, Italy) for nitrogen, carbon, and hydrogen. The polymeric structure of PKHEDA was determined by ^1^H-NMR spectroscopy in a Varian Mercury Plus 400 MHz spectrometer (Agilent, Santa Clara, CA, USA) and Attenuated Total Reflectance (ATR)–FTIR spectroscopy using a PerkinElmer Spectrum 2000 (San Francisco, CA, USA) in agreement with procedures previously reported [84].

### 2.3. Surface Activity Study of Functional Polyketones

Surface tension measurements were performed on the PKHEDA solutions at different concentration and pH values using a Lauda drop volume tensiometer TVT 1 (Hamburg, Germany). A 1.0 mL-plastic syringe was attached to a needle with a capillary radius of 1.65 mm. The temperature was set at 20 °C during the period of measurement and water density was set to 0.997 g/mL.

### 2.4. Solubility Studies of Functional Polyketones

The solubility of the functionalized polyketone was investigated in aqueous media at different pHs. A stock solution of PKHEDA was prepared at 5 × 10^−4^ M considering the mole concentration as HEDA functional groups. The stock solution was adjusted to pH 3, until visualizing the complete solubilization of the polymer. Then, the stock solution was diluted to 1 × 10^−4^ M and the pH adjusted between 1 and 12 with minimum amounts of NaOH (1 and 0.01 M) and HCl (1 and 0.01 M) for each sample and monitored with a pH-meter (Seven2GoTM pH meter, METTLER TOLEDO, Milano, Italy), calibrated at three points with 4.00, 7.00, and 10.00 buffers. The size and zeta potential of the polymeric solutions were analyzed using Dynamic Light Scattering (DLS, intensity distributions) at 25 °C. A laser beam operating at 532 nm was used and detection was performed at a fixed angle of 173° (Möbiuζ instrument WYATT TECHNOLOGY, Santa Barbara, CA, USA). Each measurement was carried out in quadruplicate. The data collection and analysis were performed with the DYNAMIC software. The accuracy of the data was confirmed by analyzing the correlograms of the polymeric suspensions (Figure S2).

### 2.5. Study of PKHEDA/TPPS Colloids

Supramolecular complexes composed by PKHEDA and TPPS were obtained by mixing the corresponding stock solutions at different pHs. The mixing protocol consisted of pouring an amount of the TPPS stock solution (1 × 10^−3^ M) dropwise under vigorous stirring into the polymer solution until the desired concentration was reached. In the systems, the polymer concentration was fixed to 1 × 10^−4^ M and the concentration of TPPS varied between 5 × 10^−5^, 1 × 10^−4^, and 2 × 10^−4^ M. Considering that the dye molecules possess 4 negative charges at their periphery, an excess of dye negative charges over the polymeric positive charges is afforded. The pH was adjusted between 2 and 11 with minimum amounts of NaOH (1 and 0.01 M) and HCl (1 and 0.01 M) for each sample. The samples were then allowed for equilibration overnight. Absorption UV–vis analyses were performed with path lengths ranging from 0.2 to 1 cm depending on the concentration of TPPS using a PerkinElmer Lambda 650 spectrophotometer (San Francisco, CA, USA) UV–vis equipment. The size and zeta potential of each sample were analyzed by Brookhaven ZetaPALS (Holtsville, NY, USA) at 25 °C. The analysis reports of the samples are shown in Figures S3–S5.

### 2.6. Ultrafiltration

Ultrafiltration experiments were performed in a batch stirred cell (50 mL) equipped with a membrane (regenerated cellulose) with an area of 13.4 cm^2^ (Amicon^®^ Merck-Millipore KGaA, Darmstadt, Germany). The supramolecular complex solutions (experimental conditions Table S1) were first characterized by DLS and UV–vis spectroscopy. Then, 10 mL of every solution was passed across the membrane (cutoff 1000 Da) under 60 bar of nitrogen pressure. When 8 mL of the initial solution has been filtered, the concentration of TPPS in the filtrate was measured by UV–vis spectroscopy (PerkinElmer Lambda 650 spectrophotometer—San Francisco, CA, USA), and then the percentage of retention (*R*%) was calculated by the following equation [86]:

(1) $$R\%=\left(1-\frac{Ap}{Ai}\right)\times 100$$

where *Ap* and *Ai* are the absorbance of TPPS in the permeate and in the initial solution, respectively, at a given wavelength. *R*% were calculated at different wavelengths (corresponding to signal maxima) and averaged.

### 2.7. Colloidal Stability

The stability of the colloidal systems composed by PKHEDA 1 × 10^−4^ M and TPPS 5 × 10^−5^ M at three different pHs (2.92, 6.29 and 10.7) was studied for 5 days. Characterization of the samples by UV–vis (PerkinElmer Lambda 650 spectrophotometer (San Francisco, CA, USA)) and fluorescence spectroscopies (Cary Eclipse Fluorescence equipment, Agilent, Santa Clara, CA, USA), DLS (zetasizer Nano-ZS (Malvern Instruments, Malvern, United Kingdom) with backscatter detection (173°), controlled by the Dispersion Technology Software (DTS 6.2, Malvern)), and scanning electron microscopy (SEM, Dual Beam FIB/SEM Helios Nano-Lab 600i, FEI, Hillsboro, OR, USA), 10 KeV accelerating voltage and variable magnification. For SEM analysis, the samples were deposited on a copper grid and were left to dry at room temperature and ambient pressure without metallic coating.

### 2.8. Calcium Alginate Sensor Beads

Calcium alginate beads containing PKHEDA/TPPS complexes were successfully formed by mixing one volume of a 3% w/v sodium alginate solution with one volume of the complexes prepared at pH between 6 and 7, so that the final PKHEDA and TPPS concentrations achieve desired values. The resulting mixtures (2.5 mL) were poured dropwise into 60 mL of 0.1 M CaCl_2_ using a 5 mL plastic syringe with a 21G needle. Calcium alginate beads were let to form during 3 min in the CaCl_2_ solutions, then filtrated over filter paper, washed with deionized water to remove excess of calcium, and then poured into water at pH 4 and pH 10. Color changes were observed by naked eye in the course of 1 h.

## 3. Result and Discussion

### 3.1. Polyketone Modification

The functionalization of the polyketone with HEDA yielded a di-carbonyl conversion of 74% as obtained from elemental analysis. The successful grafting of pendant groups on the PK was confirmed by ^1^H-NMR (Figures S6–S8) and ATR–FTIR (Figure S9) spectroscopies; the material has been successfully characterized with the help of a model compound (see experimental section).

### 3.2. Surface Tension and Self-Aggregation Process of PKHEDA

**Figure 2:**
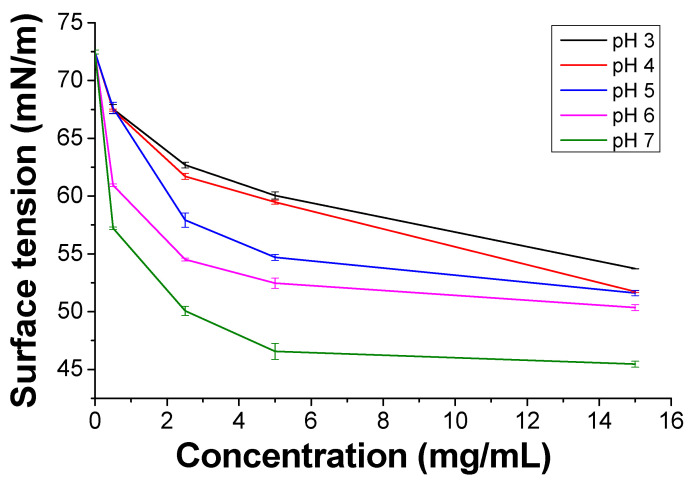
Surface tension of polyketone-*N*-(2-hydroxyethyl)ethylenediamine) (PKHEDA) as a function of the concentration at various pH values.

PKHEDA has a pH-responsive amphiphilic structure in which the hydrophilic parts consist of the secondary amine and OH pendant groups and the polymeric backbone forms the hydrophobic segment (Figure 1b). Protonation of the secondary amine makes the polymer water-soluble at acidic pH; conversely, it becomes less positively charged as the pH increases, thus yielding a more hydrophobic polymeric system [87,88]. Figure 2 shows the reduction in the surface tension of the polymer solution as a function of concentration and pH. PKHEDA exhibits a higher surface activity (lower surface tension) as the pH and concentration increase since the polymer deprotonates and the hydrophobic blocks begin to be absorbed at the water/air interface [65,66].

**Figure 3:**
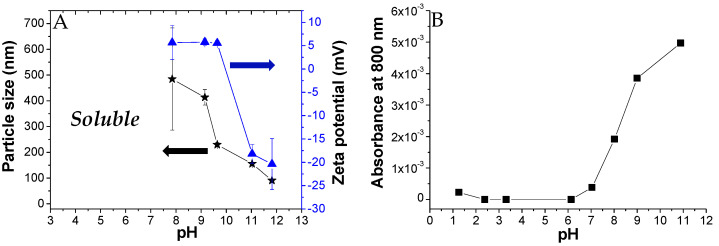
Size and zeta potential (**A**) and absorbance (**B**) of PKHEDA solutions at a concentration of 1 × 10^−4^ M of functional groups at different pH values.

We also investigated the PKHEDA self-aggregation at a concentration of 1 × 10^−4^ M of the HEDA functional groups in aqueous media. Figure 3A shows the particle size and zeta potential of colloidal systems formed as a function of pH. PKHEDA is fully soluble at acidic pH. At pH around 7.9, the system collapses, due to the increase in the hydrophobic character of the polymers associated with a decrease in the amino group protonation extent, and large (hydrodynamic diameter of around 500 nm) and polydispersed cationic particles are found showing very low positive zeta potential. In the pH range from 7.9 to 9.6, colloidal particles shrink, as deduced by a decrease in size to around 200 nm, showing a significantly lower polydispersity. Interestingly, between pH 10 and 12, an inversion on the zeta potential is observed. This is explained by the deprotonation of hydroxyl groups. Thus, before the stability of the submicron particles is lost due to deprotonation of all the ammonia groups, negative charges appear at the pendant polymer group ends, producing a further shrink of the polymer chain aggregates and negative zeta potential, high enough to ensure stability (around −20 mV). The aggregation of the polymer as a function of pH was corroborated by measurements of the absorbance of the polymer solution at 800 nm, where the functional groups do not absorb (Figure 3B). The increase in absorbance from pH 7 was assigned to the scattering generated by the formation of submicron macromolecular aggregates caused by the deprotonation of the secondary amine groups [89]. Since the size of the particles decreases with pH, but absorbance increases, it could be deduced that a higher number of particles is also obtained by increasing the pH from 7 to 12.

### 3.3. PKHEDA/TPPS Colloids

**Figure 4:**
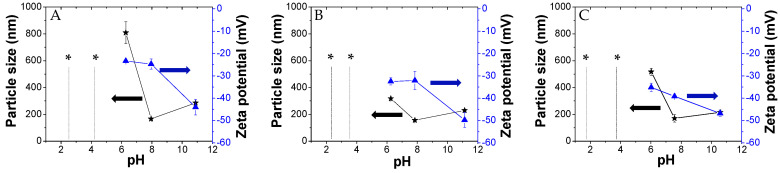
Size and zeta potential of the PKHEDA (1 × 10^−4^ M)/TPPS systems at different pHs and TPPS concentrations: (**A**) 5 × 10^−5^; (**B**) 1 × 10^−4^; (**C**) 2 × 10^−4^ M; * indicates macroparticle suspensions.

Here, we investigated the aggregation of PKHEDA/TPPS mixtures at different pHs using the ionic self-assembly method. The formation of polymeric particles with the aid of porphyrins used as cross-linkers has been reported in the literature [71,90,91]. Zhao et al. combined TPPS with poly(ethylene glycol)-block-poly(4-vinylpyridine) to synthesize polymeric complex micelles by electrostatic interactions between the pyridine moieties and the sulfonate groups of TPPS, which formed the core of the polymeric micelles [71]. Similarly, the cationic water-soluble PKHEDA at acid pH could be crosslinked with the anionic dye. Figure 4 shows particle size and zeta potential of three systems composed of the polymer at a fixed concentration of 1 × 10^−4^ M and TPPS at different concentration ratios (PKHEDA/TPPS of 1/0.5, 1/1, and 1/2) as a function of the pH. Note that as TPPS may be found mostly in two forms of different net charges, either the di-anionic form of the dye (H_4_TPPS^2−^) or the tetra-anionic form H_2_TPPS^4−^ (see Figure 1), the total amount of TPPS negative charges equals or is larger than the total amount of polymeric positive charges. Pristine diluted aqueous solutions of TPPS show transition between H_4_TPPS^2−^ and H_2_TPPS^4−^ at pH around 4.3 (see Figure S10). This value changes with concentration and the presence of other species in solution. At pH lower than 4, PKHEDA is highly positively charged due to the protonation of most of the secondary amines. Thus, polymer and dye interact by electrostatic interactions, neutralizing each other, and producing flocculating systems that easily collapse, and that can be easily resuspended upon shaking. Gradually increasing the pH, stable submicron particles are found for all systems, showing negative zeta potential from pH 6 to 11. In this pH range, TPPS molecules are mostly found as tetra-anionic H_2_TPPS^4−^. This form of the dye is more hydrophobic than its partner, H_4_TPPS^2−^, due to the fact that the latter presents six charged groups, four negative at its periphery, and two positive at its geometrical center. Thus, H_2_TPPS^4−^ is more prone to undergo preferential solvation with the polymer segments [84], so that the amphiphilic polymer incorporates a relatively large amount of dye molecules in its domain that affords an excess of negative charges over the polymeric positive charges (which, in addition, are decreasing as the pH increases) stabilizing submicron particles. Hydrophobic interactions, π–π interactions, and intermolecular hydrogen bonding are responsible for the molecular assembling.

**Figure 5:**
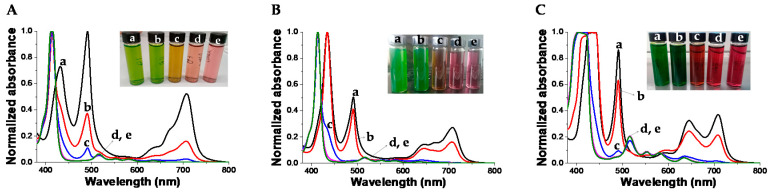
Normalized UV–vis spectra of TPPS at different concentration and pH in the presence of PKHEDA 1 × 10^−4^ M. (**A**) TPPS 5 × 10^−5^ M and pHs: (a) 2.57, (b) 4.07, (c) 6.28, (d) 7.95, (e) 10.89. (**B**) TPPS 1 × 10^−4^ M and pHs: (a) 2.33, (b) 3.72, (c) 6.26, (d) 7.85, (e) 11.13. (**C**) TPPS 2 × 10^−4^ M pHs: (a) 2.55, (b) 4.66, (c) 6.02, (d) 7.56, (e) 10.58.

At acidic pH, H_4_TPPS^2−^ in the presence of PKHEDA are mostly found as J-aggregates. This can be seen in Figure 5, by the green color of the corresponding suspensions, and the associated UV–vis spectra, showing the characteristic Soret band at 490 nm (see Figure 5) [92,93,94]. The Soret band corresponding to the monomeric form of the di-anionic dye at 434 nm is also found, as well as the Q-bands centered at 640 and 700 nm associated with the D4h symmetry group of H_4_TPPS^2−^ [70]. It is worth mentioning that suspended floccules also possess green color after settling for 10 min at pHs between 2 and 4. On the other hand, at basic pH, the submicron particles show H_2_TPPS^4−^ dispersed as monomers. This can be seen by the pink-red color of the corresponding suspensions, associated with the Soret band of the monomeric tetra-anionic form of the dye at around 410–414 nm and Q-bands centered at 516, 553, 580, 635 nm, four bands justified by the D2h symmetry group of H_2_TPPS^4−^.

Summarizing, we can observe that the PKHEDA/TPPS systems form different types of self-organized structures. At acidic pH, the system collapses due to the charge neutralization of the polymer by the TPPS in its H_4_TPPS^2−^ form, which additionally self-aggregates as J-aggregates. As the pH is increased, the systems undergo structural reorganization to submicron-sized colloids of negative zeta potential, showing the polymer with swollen H_2_TPPS^4−^ monomeric species.

**Figure 6:**
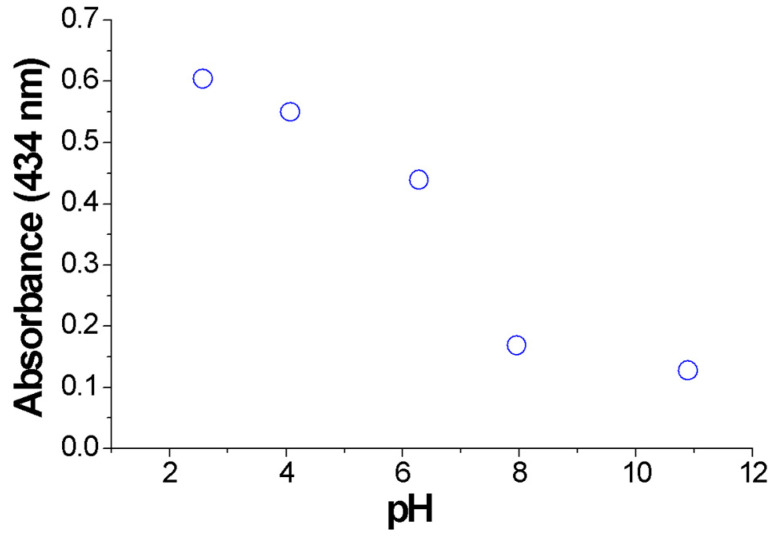
Absorbance variation at 434 nm of the colloids (PKHEDA 1 × 10^−4^ M/TPPS 5 × 10^−5^ M) as a function of pH.

Transition between the di-anionic and the tetra-anionic form of the dye occurs for the pristine dye in a short range of pH (see Figure S10). However, the presence of the polymer affords different chemical environment and exchange kinetics, so that the transition looks smoother, occurring in a wider pH range. The brown color obtained at pH 6 arises from the contribution of both the tetra-anionic H_2_TPPS^4−^ and residual H_4_TPPS^2−^ J-aggregates, as witnessed in the respective UV-vis bands. Figure 6 shows the absorbance variation at 434 nm of the colloidal systems formed by PKHEDA and TPPS at a concentration of 1 × 10^−4^ and 5 × 10^−5^ M, respectively. The system shows a significant and almost linear variation in absorbance as the pH changes from acidic to basic.

### 3.4. Ultrafiltration Studies

**Figure 7:**
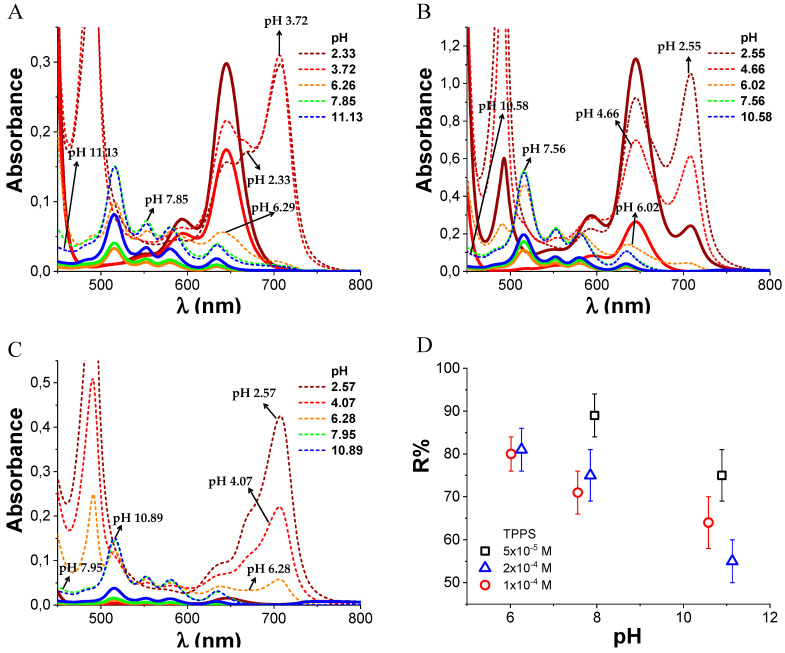
Absorbance spectra of TPPS/PKHEDA mixtures at different pH at 1 × 10^−4^ M PKHEDA in the initial suspension (dotted line) and in the filtrate (continuous line). TPPS concentration 1 × 10^−4^ M (**A**), 2 × 10^−4^ M (**B**) and 5 × 10^−5^ M (**C**). Plot of R% at different pHs and TPPS concentrations (**D**).

We studied by ultrafiltration the retention of TPPS by PKHEDA in water at different pHs to qualitatively evaluate the noncovalent interaction of the systems. UV–vis analysis of solutions in the filtrate (Figure 7A–C) shows that the filtered dye does not self-aggregate in the form of J-aggregates, witnessed by the shifting of both the Soret and the Q-bands to higher energies, corresponding to the monomeric di-anionic species. Although the dye at these conditions is more diluted, this observation is consistent with previous observations that show that J-aggregates are induced by a high local concentration of the dye around complementary charged polymers [67]. Retention values (%) of the dye in the retentate containing the polymer/dye complex stand above 50% (see Figure 7D). Note that retention tends to decrease at higher pH but is still quite significant. Considering that at pH over 10 both PKHEDA and TPPS are negatively charged, the strong contribution of preferential solvation [84] is emphasized, as well as π-π stacking [67], hydrophobic interactions [95], and hydrogen bonding [42], thus favoring the formation of supramolecular assemblies, even overcoming charge repulsion. The highest retention values are given at lower pHs, so that most of the dye molecules remain bound to the polymer, and for TPPS 5 × 10^−5^ M, the dye is not found in the filtrate. The high retention of TPPS by PKHEDA by different supramolecular interactions points at the possibility for PKHEDA surfactant polymers being applied for the encapsulation of aromatic drugs and the retention of charged aromatic pollutants for water remediation.

### 3.5. Colloid Stability

**Figure 8:**
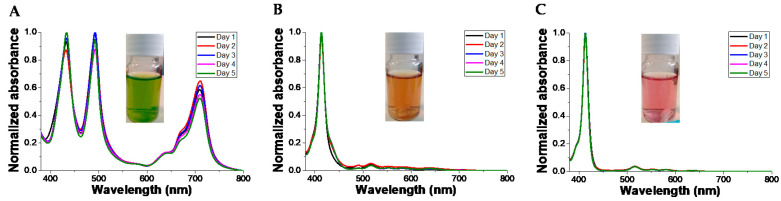
Normalized UV–vis spectra of TPPS at a concentration of 5 × 10^−5^ M and different pH in the presence of PKHEDA 1 × 10^−4^ M. pHs: (**A**) 2.92, (**B**) 6.29, (**C**) 10.70.

We studied the stability of the colloidal system formed by TPPS at the concentration of 5 × 10^−5^ M and PKHEDA 1 × 10^−4^ M at different pHs for 5 days. As can be seen in Figure 8 and Figure S11, changes in the UV–vis behavior of the suspensions are modest. However, DLS witnesses the tendency to flocculate of the samples, even at pH 6.92 and 10.70, although floccules easily remain hydrated and suspended. Thus, correlograms obtained from day 2 lose accuracy, and we obtain data quality beyond the limit of confidence according to the equipment software.

**Figure 9:**
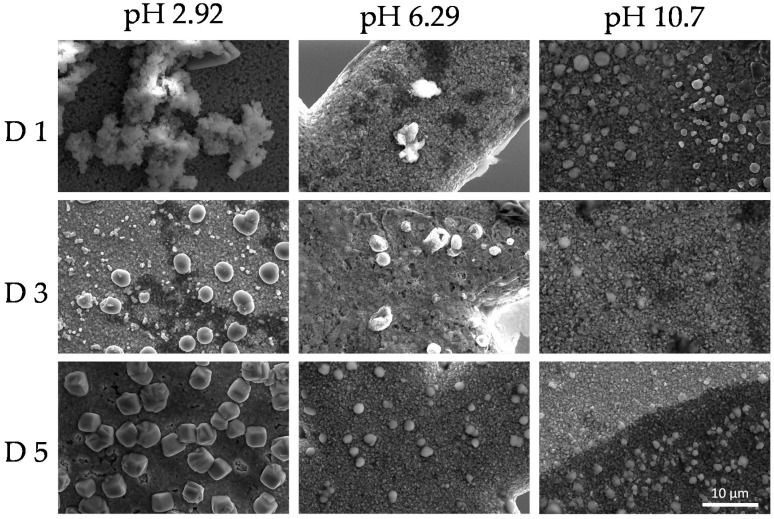
Scanning electron microscopy (SEM) micrographs at a magnification ×5000 of the colloids (PKHEDA 1 × 10^−4^ M/TPPS 5 × 10^−5^) at three different pH (2.92, 6.29, and 10.70) during five days (day 1 = D1, day 3 = D3, day 5 = D5).

**Figure 10:**
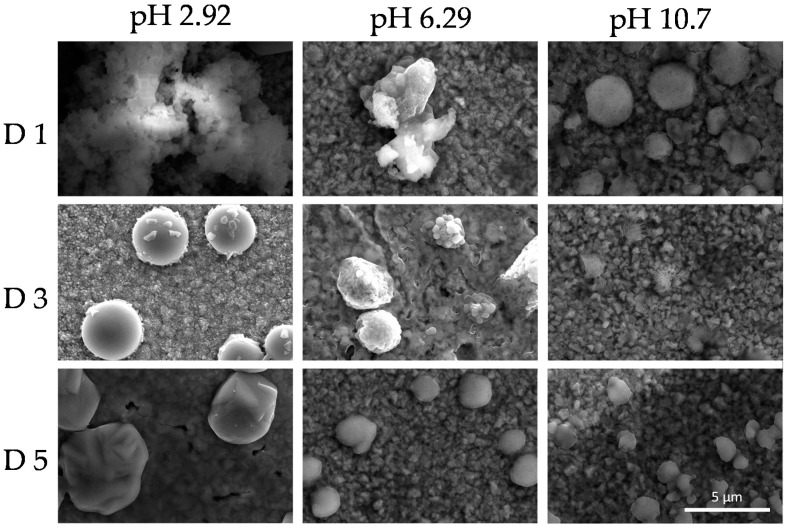
Scanning electron microscopy (SEM) micrographs at a magnification ×15,000 of the colloids (PKHEDA 1 × 10^−4^ M/TPPS 5 × 10^−5^) at three different pH (2.92, 6.29, and 10.70) during five days (day 1 = D1, day 3 = D3, day 5 = D5).

This phenomenon indicates the reorganization of the colloidal system as a function of time. SEM images shown in Figure 9 and Figure 10 revealed dry deposits of the colloidal supramolecular structures at two different magnifications carried out at day 1, 3, and 5. The collapse of the complexes upon drying produces fractal-like globular aggregates which afford texture to the deposits. However, over 5 days, particles with the form of rounded cubes seem to be formed. These kinds of particles have been described in the literature [96] for polymeric porphyrin frameworks, revealing a more crystalline organization of the complexes.

### 3.6. Applications

**Figure 11:**
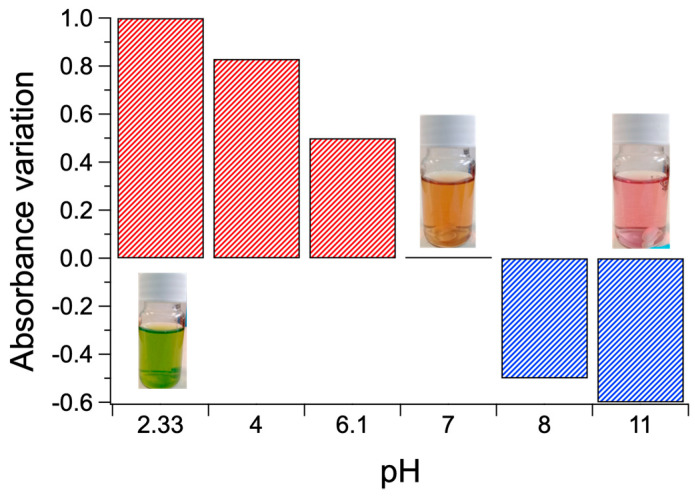
Positive (red) and negative (blue) absorbance variations from neutrality at 434 nm of the PKHEDA 1 × 10^−4^ M/TPPS 5 × 10^−5^ M colloids and corresponding color changes of the respective water solutions.

The materials appear sensitive to pH modification, i.e., displaying substantial absorbance variations from neutrality. Notably, positive and negative absorbance variations occur at acidic and basic pHs, respectively (Figure 11), flanked by distinct color changes of solutions from orange to green (acidification) and from orange to pink (basification).

**Figure 12:**
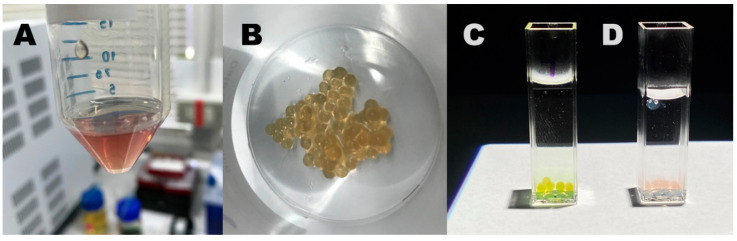
(**A**) Sodium alginate (1.5% w/v) containing the PKHEDA 2 × 10^−4^ M/TPPS 1 × 10^−4^ M colloids at pH 6.0; (**B**) freshly prepared calcium alginate beads containing the PKHEDA/TPPS complex; the calcium alginate beads were poured in water at pH 4 (**C**) and 10 (**D**).

Such phenomena clearly suggest applications in the field of optical sensors and smart packaging to determine, for instance, potential deterioration of foods or drugs that experience degradation with pH changes. Moreover, due to the relatively smooth transition in terms of absorption at specific wavelengths in reply to pH shift, such materials should also act as pH transducers in specific pH sensor systems. As an example, it could be immobilized in a suitable cross-linked hydro-gel matrix to implement a sensitive and cheap pH sensor with colorimetric (LED-photodiode based) red-out (see Figure 12). Moreover, if applied to a fiber optic architecture [97], it is possible to obtain a miniaturized pH probe able to perform continuous measurement at low energy consumption with the capability of accurate measurement in biological pH range.

Such kinds of optical sensors could find important applications in water environmental monitoring scenarios, since the pH is one of most important indicator of water quality (wastewater and hazardous chemicals can significantly alter the natural pH of freshwaters). Finally, miniaturized, low cost and accurate pH sensors, can find countless applications also in the industrial processes including semiconductors, food, drugs, cement and textile, pharma, consumer goods, and so on [97].

## 4. Conclusions

We have reported the synthesis of supramolecular submicron structures promoted by the effective combination of TPPS with a pH-responsive surfactant PK derivative bearing a secondary amine and hydroxyl as pendant groups. The amphiphilic properties of the polymer are the result of the hydrophobic character of the polymer backbone and the basic and weak-acid properties of the pendant group. The polymer was able to interact with TPPS via supramolecular interactions with retention capability higher than 50% depending on the pH. At acidic pH, the polymer is rather hydrophilic and water-soluble due to a high degree of amino groups protonation, and upon interaction with TPPS induces J-aggregates of the dye, and flocculation of the systems in the form of hydrated particles that remain easily suspended by shaking. At increasing pHs, the polymer loses positive charges due to deprotonation, and becomes more hydrophobic, as TPPS does. Then, dye molecules are swollen in the polymer domain, so that an excess of negative charges stabilize shrunk, submicron particles of the complex system. At pH over 10, the polymer itself presents negative charges due to deprotonation of the terminal hydroxyl group. Despite this fact, interaction with the also negatively charged dye occurs, and submicron colloidal particles containing both molecules are formed. The self-assembled structures showed dynamic color change in the visible region. The size, zeta potential, and color of the supramolecular assemblies were readily tuned by varying the pH of the solution and the concentration of TPPS. The visible and smooth change in the color suggests that the prepared assembled systems might be used in the fabrication of pH optical sensors.

## Supplementary Materials

The following are available online at https://www.mdpi.com/2073-4360/12/9/2017/s1. Section S1: Model compound synthesis, Section S2: Polyketone modification, Figure S1: Schematic structure of J-aggregates (left) and H-aggregates (right) formed by TPPS, Figure S2: Correlograms of the PKHEDA 1 × 10^−4^ M polymeric suspensions, Figure S3: Size analysis report of the PKHEDA 1 × 10^−4^ M/ TPPS 5 × 10^−5^ system at different pHs (A) 6.28, (B) 7.95, (C) 10.89, Figure S4: Size analysis report of the PKHEDA 1 × 10^−4^ M/TPPS 1 × 10^−4^ system at different pHs (A) 6.26, (B) 7.85, (C) 11.13, Figure S5: Size analysis report of the PKHEDA 1 × 10^−4^ M/TPPS 2 × 10^−4^ system at different pHs (A) 6.02, (B) 7.56, (C) 10.58, Figure S6: ^1^H-NMR spectrum of pyrrol-HEDA model compound used as reference to assign the peaks to the polymer, Figure S7: ^1^H NMR spectra of PK before (red dashed line) and after (solid black line) modification with HEDA, Figure S8. ^1^H NMR spectrum of PK functionalized with HEDA and its respective integrals, Figure S9. ATR-FTIR spectra of PK before (red dashed line) and after (solid blue line) modification with HEDA. Notice the hydrogen bonding peak-band around 3300 cm^−1^), Figure S10. (A) Transition pH of TPPS at a concentration of 1 × 10^−6^ M. The absorbance of the TPPS band at 434 nm was used to estimate the transition pH in a range of pH from 1 to 9 that reveals the protonation of the pyrrole groups. (B) Fitting data using the Boltzmann sigmoidal curve and further derivation to find the transition pH of TPPS at 4.35, Figure S11: Fluorescence spectra of TPPS at a concentration of 5 × 10^−5^ M and different pH in the presence of PKHEDA 1 × 10^−4^ M. pHs: (A) 2.92, (B) 6.29, (C) 10.7. Excitation slit 5 nm and emission slit 10 nm. Excitation wavelength 430 nm, Table S1: Ultrafiltration experimental conditions.
