# Supplementary material for: pH-Responsive Polyketone/5,10,15,20-Tetrakis-(Sulfonatophenyl)Porphyrin Supramolecular Submicron Colloidal Structures
Source: Polymers (Basel). 2020 Sep 3;12(9):2017. doi: 10.3390/polym12092017 (PMC7563153; doi:10.3390/polym12092017)
Supplement: Supplementary file 1 [file polymers-12-02017-s001.pdf]

Article

pH-Responsive Polyketone/5,10,15,20-Tetrakis-(Sulfonatophenyl)Porphyrin Supramolecular Submicron Colloidal Structures

Esteban Araya-Hermosilla [1,*], Ignacio Moreno-Villoslada [2,*], Rodrigo Araya-Hermosilla [3], Mario E. Flores [2], Patrizio Raffa [4], Tarita Biver [5], Andrea Pucci [6], Francesco Picchioni [4] and Virgilio Mattoli [1,*]

[1] Center for Micro-BioRobotics, Istituto Italiano di Tecnologia Viale Rinaldo Piaggio 34, 56025 Pontedera (PI), Italy
[2] Laboratorio de Polímeros, Instituto de Ciencias Químicas, Facultad de Ciencias, Universidad Austral de Chile, Valdivia 5090000, Chile; mario.flores@uach.cl
[3] Programa Institucional de Fomento a la Investigación, Desarrollo e Innovación, Universidad Tecnológica Metropolitana, Ignacio Valdivieso 2409, P.O. Box 8940577, San Joaquín, Santiago 8940000, Chile; rodrigo.araya@utem.cl
[4] Department of Chemical Product Engineering, ENTEG, University of Groningen, Nijenborgh 4, 9747AG Groningen, The Netherlands; p.raffa@rug.nl (P.R.); f.picchioni@rug.nl (F.P.)
[5] Dipartimento di Farmacia, Università di Pisa, Via Bonanno 6, 56126 Pisa, Italy; tarita.biver@unipi.it
[6] Dipartimento di Chimica e Chimica Industriale, Università di Pisa, Via Moruzzi 13, 56124 Pisa, Italy; andrea.pucci@unipi.it
* Correspondence: esteban.araya@iit.it (E.A.-H.); imorenovilloslada@uach.cl (I.M.-V.); virgilio.mattoli@iit.it (V.M.); Tel.: +39-050-883417 (E.A.-H.); +56-(63)-2293520 (I.M.-V.); +39-050-883417 (V.M.)

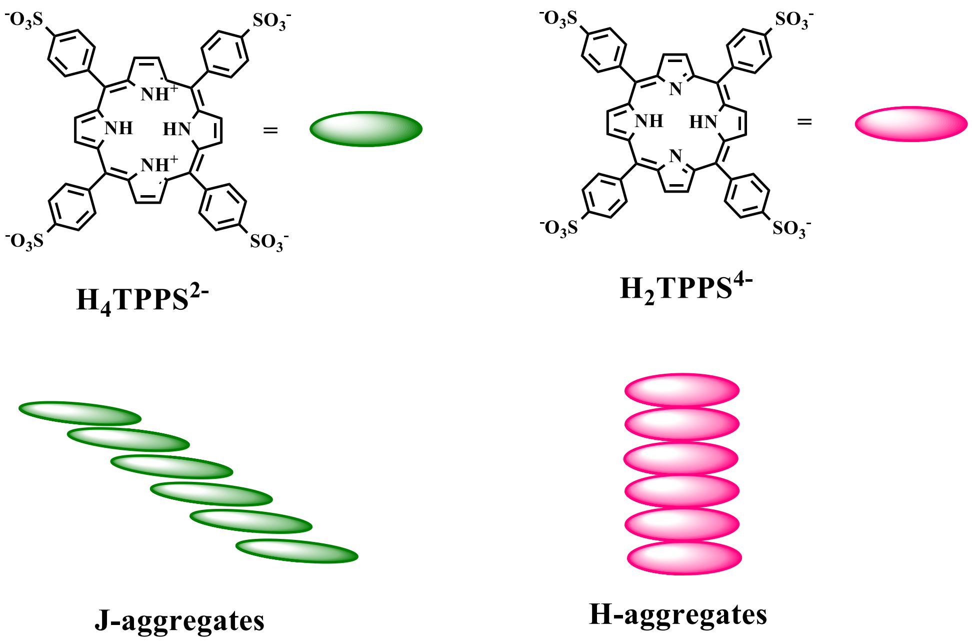

Figure S1. Schematic structure of J-aggregates (left) and H-aggregates (right) formed by TPPS.

Section S1: Model compound synthesis

As described previously by our group [1], a model compound was synthetized and use as a reference to characterize the complex structure of the polymer. The model compound system was synthetized by mixing a one to one ratio between 2,5-hexadione and HEDA in a round bottom flask provided with a magnetic stirrer and reflux. The reaction was set under vigorous stirring at 100 °C for 4 h using a Heat-On™ Block System. The product was analyzed by ^{1}H NMR (Varian Mercury Plus 400 MHz spectrometer) using CDCl3 as solvent. δ = 2.2 ppm (s, 6 H), 2.7 ppm (t, 2H, J = 6.9 Hz), 2.9 ppm (t, 2H, J = 6.9 Hz), 3.6 ppm (t, 2H, J = 6.9 Hz) 3.9 ppm (t, 2H, J = 6.9 Hz), 5.8 ppm (s, 2H).

Section S2: Polyketone modification

The PK was preheated to the liquid state at 100 °C for a few min. Then, HEDA was added dropwise to the reactor in the first 30 min. The stirring speed was set at 400 rpm during 4 h. During the reaction, the color of the reaction mixture changed progressively from light yellow to a range of brown due to pyrrole formation as polymer backbone. The product was cooled down to room temperature and dissolved in chloroform for purification by the solvent/solvent extraction technique using brine as co-solvent. The process was repeated three times to remove unreacted amine molecules. The resulting polymer solution was dried at reduced pressure and transferred to a vacuum oven at 50 °C for 24 h for complete dryness. To avoid hydration, the samples were sealed in brown glass vials and stored at 6 °C for further characterization. Finally, the polymers were analyzed by ^{1}H-NMR and ATR-FT-IR. The carbonyl conversion was defined as the molar fraction of 1,4-dicarbonyl units converted via the Paal-Knorr reaction (x). It was calculated by elemental analysis EA (Elementar Vario Micro Cube) as follows.

$$x = \frac{NM_c}{nM_N + N(M_c - M_p)} \quad (1)$$

where N is the nitrogen content per g, M_N represents the atomic mass of nitrogen (14 g/mol), n is the number of nitrogen atoms of the converted 1,4-dicarbonyl segment (2 units for HEDA), M_p is the molecular weight of the converted 1,4-dicarbonyl segment (194 g/mol for HEDA), and M_c is the molecular weight of the non-converted 1,4-dicarbonyl segment (126 g/mol) of PK. M_p and M_c were calculated taking into account the incorporation of ethylene and propylene in the copolymers at a ratio of 1:1.

In order to adjust the stoichiometry of the functional groups, a polymer molecular weight (PMW) of 236 g/mol of aliphatic amino groups (or hydroxyl groups) was considered, related with x of 0.74. This is calculated by the following formula.

$$PMW = \frac{xM_p + yM_c}{x} \quad (2)$$

where y is the fraction of non-converted 1,4-dicarbnyl groups, provided by $x + y = 1$.

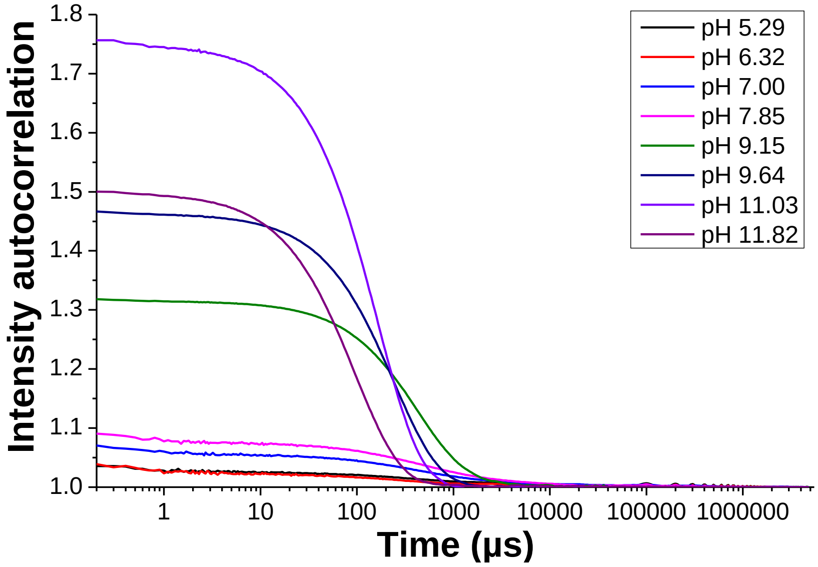

Figure S2. Correlograms of the PKHEDA (1 × 10^{-4} M) polymeric suspensions.

A

000005ph6.28 (Combined)
Nov 12, 2019 08:04:48

Effective Diameter: 825.1 nm
Polydispersity: 0.324
Avg. Count Rate: 301.4 kcps
Baseline Index: 8.0/ 98.08%
Elapsed Time: 00:09:00

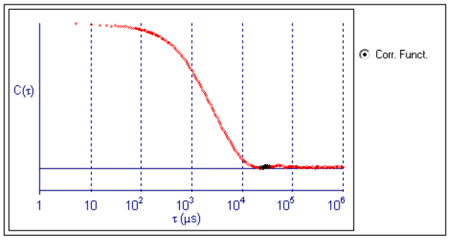

Run	Eff. Diam. (nm)	Half Width (nm)	Polydispersity	Baseline Index
1	950.8	452.0	0.226	5.5/ 97.84%
2	807.1	458.2	0.322	9.9/ 97.12%
3	668.4	406.3	0.369	9.9/ 99.28%
Mean	808.7	438.8	0.306	8.4/ 98.08%
Std. Error	81.5	16.4	0.042	1.5/ 0.63
Combined	825.1	469.7	0.324	8.0/ 98.08%

B

000005ph7.95 (Combined)
Nov 12, 2019 08:19:29

Effective Diameter: 169.8 nm
Polydispersity: 0.285
Avg. Count Rate: 118.5 kcps
Baseline Index: 7.5/ 94.67%
Elapsed Time: 00:09:00

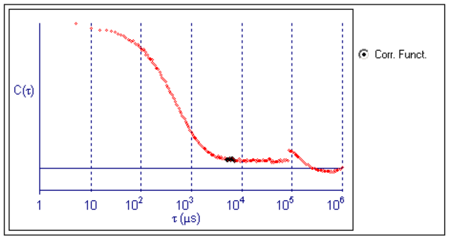

Run	Eff. Diam. (nm)	Half Width (nm)	Polydispersity	Baseline Index
1	178.9	97.5	0.297	8.0/ 95.45%
2	165.8	88.7	0.286	9.0/ 94.76%
3	154.7	84.5	0.298	8.5/ 93.79%
Mean	166.5	90.3	0.294	8.5/ 94.67%
Std. Error	7.0	3.8	0.004	0.3/ 0.48
Combined	169.8	90.7	0.285	7.5/ 94.67%

C

000005ph10.89 (Combined)
Nov 12, 2019 08:36:05

Effective Diameter: 269.0 nm
Polydispersity: 0.439
Avg. Count Rate: 79.0 kcps
Baseline Index: 8.3/ 94.48%
Elapsed Time: 00:09:00

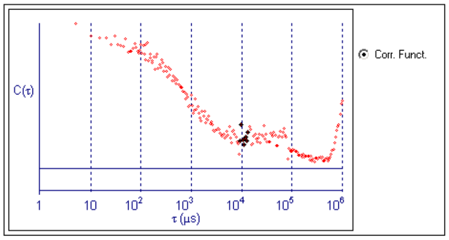

Run	Eff. Diam. (nm)	Half Width (nm)	Polydispersity	Baseline Index
1	299.4	249.1	0.692	8.9/ 92.27%
2	277.6	190.4	0.470	8.5/ 94.34%
3	279.3	198.7	0.506	9.1/ 96.84%
Mean	285.4	212.7	0.556	8.9/ 94.48%
Std. Error	7.0	18.3	0.069	0.2/ 1.32
Combined	269.0	178.1	0.439	8.3/ 94.48%

Figure S3. Size analysis report of the PKHEDA (1 × 10^{-4} M)/ TPPS (5 × 10^{-5}) system at different pHs (**A**) 6.28, (**B**) 7.95, (**C**) 10.89.

A

0.0001ph6.26 (Combined)
Nov 4, 2019 13:38:27

Effective Diameter: 317.9 nm
Polydispersity: 0.316
Avg. Count Rate: 375.2 kcps
Baseline Index: 9.1/ 98.24%
Elapsed Time: 00:09:00

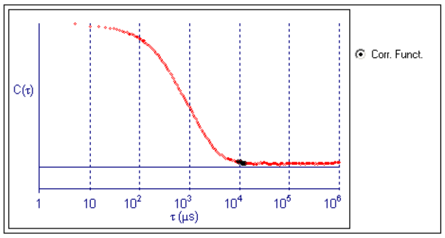

Run	Eff. Diam. (nm)	Half Width (nm)	Polydispersity	Baseline Index
1	306.8	176.9	0.332	9.9/ 98.56%
2	322.1	176.6	0.301	9.8/ 96.88%
3	323.8	183.4	0.321	8.9/ 99.28%
Mean	317.6	178.9	0.318	9.5/ 98.24%
Std. Error	5.4	2.2	0.009	0.3/ 0.71
Combined	317.9	178.8	0.316	9.1/ 98.24%

B

0.0001ph7.85 (Combined)
Nov 4, 2019 13:54:15

Effective Diameter: 159.4 nm
Polydispersity: 0.303
Avg. Count Rate: 169.2 kcps
Baseline Index: 9.2/ 93.18%
Elapsed Time: 00:09:00

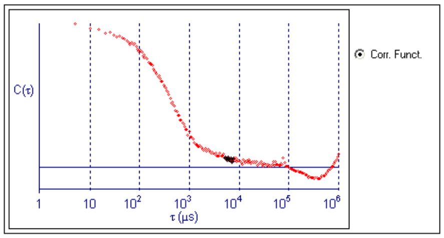

Run	Eff. Diam. (nm)	Half Width (nm)	Polydispersity	Baseline Index
1	161.9	97.4	0.362	9.3/ 94.84%
2	158.6	88.9	0.314	8.8/ 93.86%
3	148.1	87.5	0.349	9.5/ 90.84%
Mean	156.2	91.3	0.342	9.2/ 93.18%
Std. Error	4.2	3.1	0.014	0.2/ 1.20
Combined	159.4	87.8	0.303	9.2/ 93.18%

C

0.0001ph11.13 (Combined)
Nov 4, 2019 14:14:01

Effective Diameter: 233.7 nm
Polydispersity: 0.419
Avg. Count Rate: 125.1 kcps
Baseline Index: 9.1/ 93.04%
Elapsed Time: 00:09:00

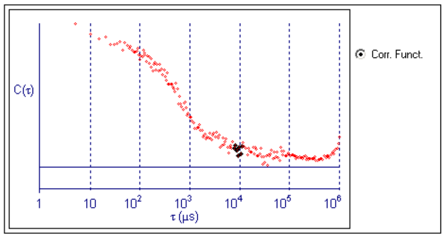

Run	Eff. Diam. (nm)	Half Width (nm)	Polydispersity	Baseline Index
1	245.7	164.8	0.450	8.8/ 95.32%
2	230.2	163.6	0.505	9.3/ 91.72%
3	212.8	147.7	0.482	9.2/ 92.10%
Mean	229.6	158.7	0.479	9.1/ 93.04%
Std. Error	9.5	5.5	0.016	0.2/ 1.14
Combined	233.7	151.3	0.419	9.1/ 93.04%

Figure S4. Size analysis report of the PKHEDA (1 × 10^{-4} M)/TPPS (1 × 10^{-4}) system at different pHs (**A**) 6.26, (**B**) 7.85, (**C**) 11.13.

A

00002ph6.02 (Combined)
Nov 6, 2019 11:35:14

Effective Diameter: 512.0 nm
Polydispersity: 0.399
Avg. Count Rate: 369.1 kcps
Baseline Index: 8.8/ 98.68%
Elapsed Time: 00:09:00

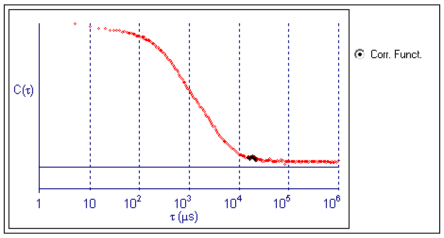

Run	Eff. Diam. (nm)	Half Width (nm)	Polydispersity	Baseline Index
1	533.3	338.7	0.403	8.0/100.00%
2	465.4	297.5	0.409	8.5/ 98.44%
3	553.7	348.2	0.395	9.9/ 97.60%
Mean	517.5	328.1	0.402	8.8/ 98.68%
Std. Error	26.7	15.6	0.004	0.6/ 0.70
Combined	512.0	323.5	0.399	8.8/ 98.68%

B

00002ph6.02 (Combined)
Nov 6, 2019 11:49:25

Effective Diameter: 162.7 nm
Polydispersity: 0.256
Avg. Count Rate: 432.4 kcps
Baseline Index: 3.6/ 96.41%
Elapsed Time: 00:09:00

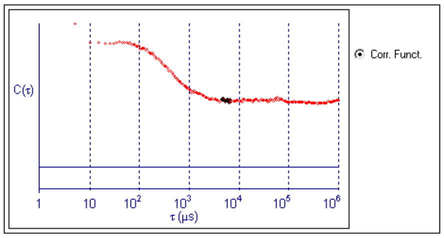

Run	Eff. Diam. (nm)	Half Width (nm)	Polydispersity	Baseline Index
1	145.4	82.1	0.319	9.6/ 94.62%
2	150.4	86.6	0.331	9.6/ 95.34%
3	214.0	66.0	0.095	3.9/ 99.28%
Mean	169.9	78.2	0.248	7.7/ 96.41%
Std. Error	22.1	6.2	0.077	1.9/ 1.45
Combined	162.7	82.3	0.256	3.6/ 96.41%

C

00002ph10.58 (Combined)
Nov 6, 2019 12:06:44

Effective Diameter: 232.6 nm
Polydispersity: 0.376
Avg. Count Rate: 286.4 kcps
Baseline Index: 1.0/ 97.68%
Elapsed Time: 00:09:00

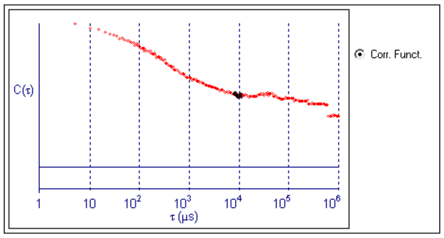

Run	Eff. Diam. (nm)	Half Width (nm)	Polydispersity	Baseline Index
1	242.4	151.1	0.389	6.2/ 97.60%
2	216.7	138.2	0.407	8.4/ 96.88%
3	185.5	120.0	0.418	9.5/ 98.56%
Mean	214.9	136.4	0.405	8.0/ 97.68%
Std. Error	16.4	9.0	0.009	1.0/ 0.49
Combined	232.6	142.6	0.376	1.0/ 97.68%

Figure S5. Size analysis report of the PKHEDA (1×10^{-4} M)/TPPS (2×10^{-4}) system at different pHs (**A**) 6.02, (**B**) 7.56, (**C**) 10.58.

Table S1. Ultrafiltration experimental conditions

Concentration PKHEDA (M functional groups)	Concentration TPPS (M)	pH	Solution (mL)
1×10^{-4}	5×10^{-5}	2.57	10
1×10^{-4}	5×10^{-5}	4.07	10
1×10^{-4}	5×10^{-5}	6.28	10
1×10^{-4}	5×10^{-5}	7.95	10
1×10^{-4}	5×10^{-5}	10.89	10
1×10^{-4}	1×10^{-4}	2.33	10
1×10^{-4}	1×10^{-4}	3.72	10
1×10^{-4}	1×10^{-4}	6.26	10
1×10^{-4}	1×10^{-4}	7.85	10
1×10^{-4}	1×10^{-4}	11.13	10
1×10^{-4}	2×10^{-4}	2.55	10
1×10^{-4}	2×10^{-4}	4.66	10
1×10^{-4}	2×10^{-4}	6.02	10
1×10^{-4}	2×10^{-4}	7.56	10
1×10^{-4}	2×10^{-4}	10.58	10

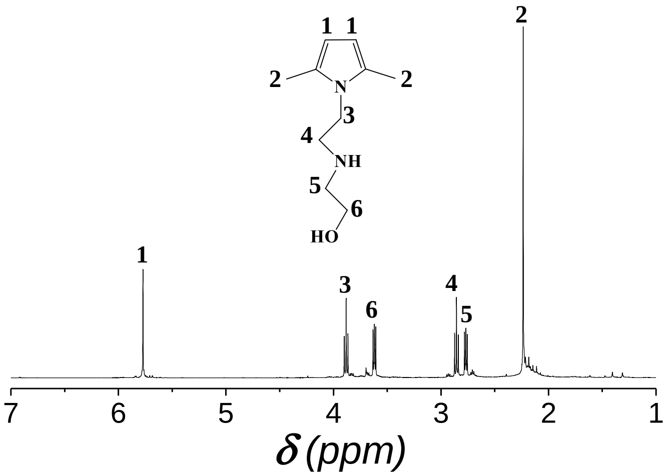

Figure S6. ^{1}H-NMR spectrum of pyrrol-HEDA model compound used as reference to assign the peaks to the polymer.

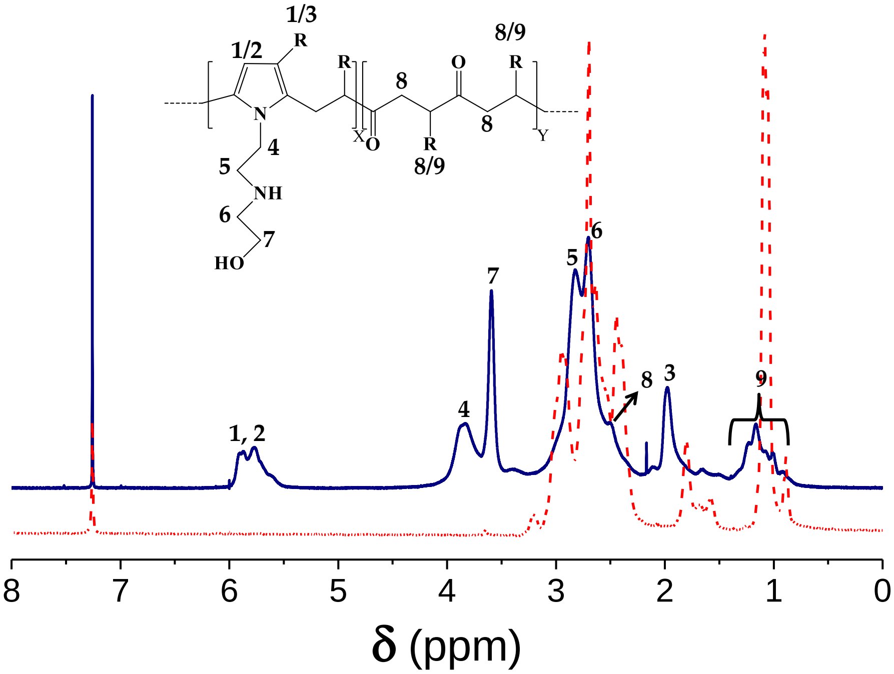

Figure S7. ¹H NMR spectra of PK before (red dashed line) and after (solid black line) modification with HEDA

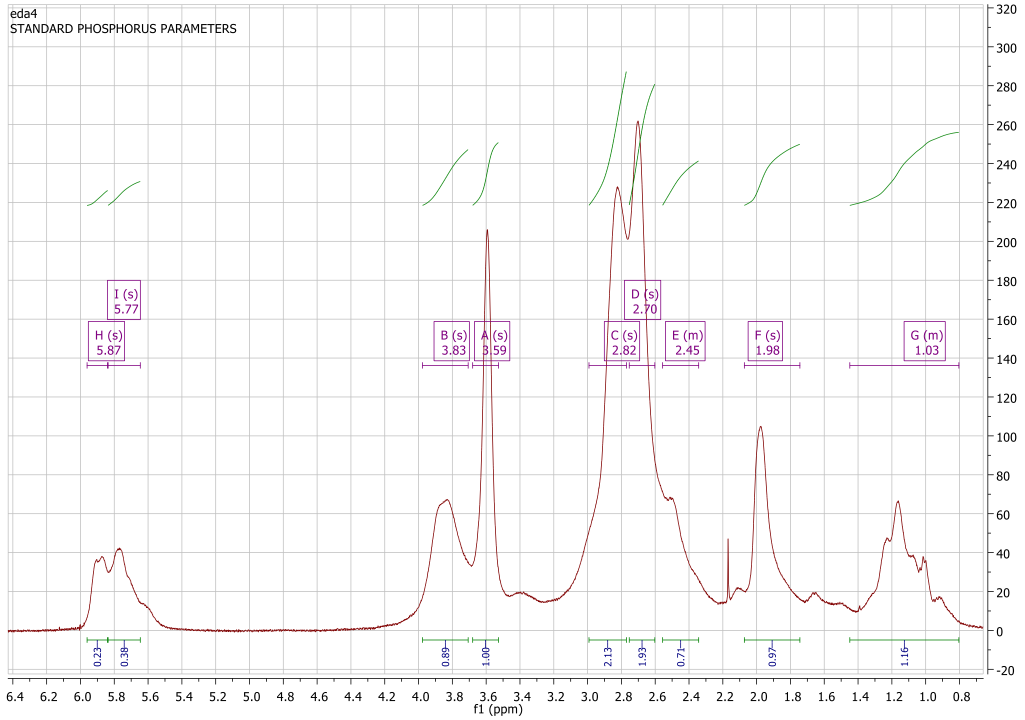

Figure S8. ¹H NMR spectrum of PK functionalized with HEDA and its respective integrals.

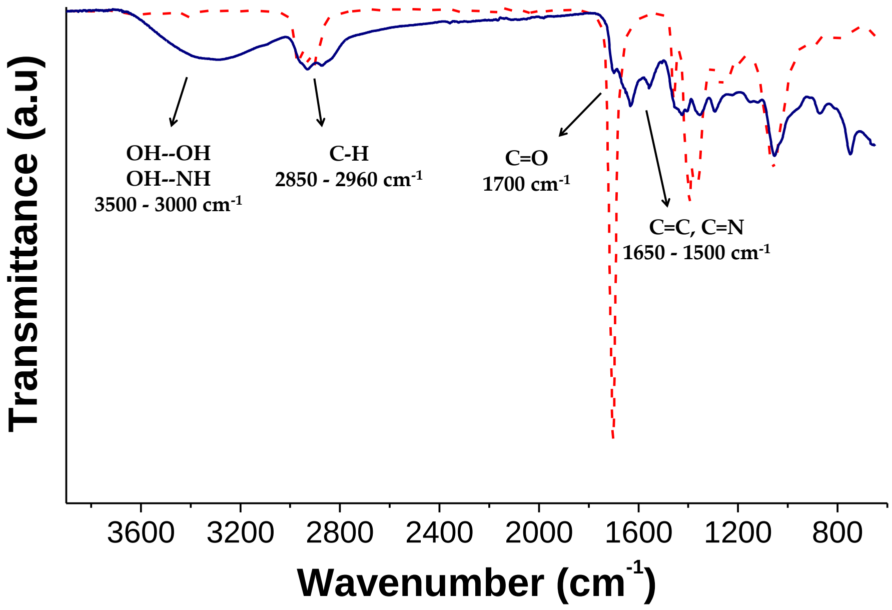

Figure S9. ATR-FTIR spectra of PK before (red dashed line) and after (solid blue line) modification with HEDA. Notice the hydrogen bonding peak-band around 3300 cm^{-1})

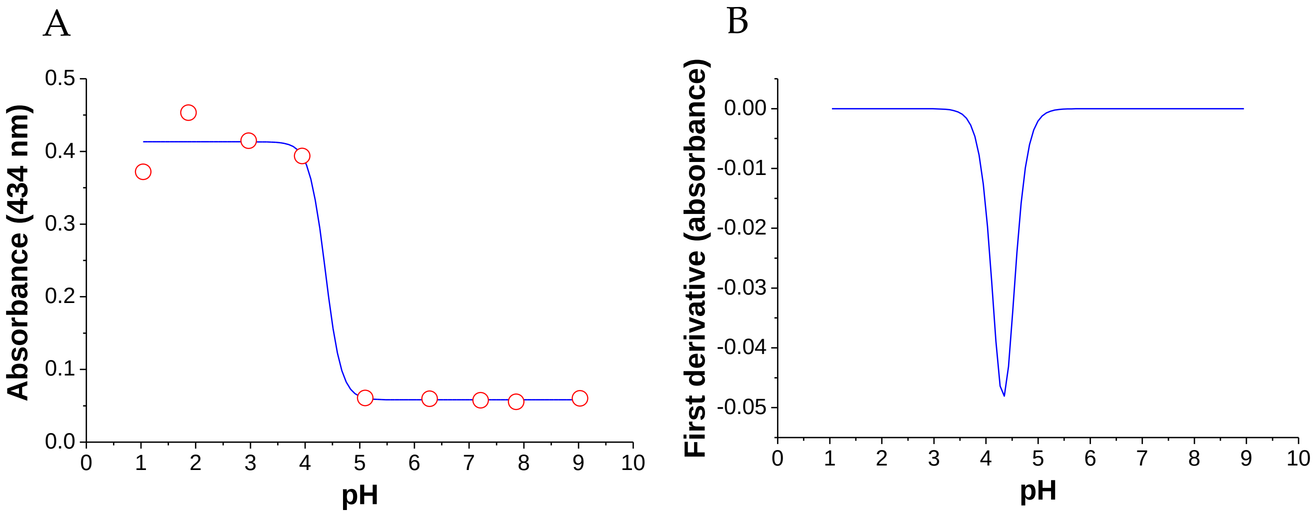

Figure S10. (**A**) Transition pH of TPPS at a concentration of 1 × 10^{-6} M. The absorbance of the TPPS band at 434 nm was used to estimate the transition pH in a range of pH from 1 to 9 that reveals the protonation of the pyrrole groups. (**B**) Fitting data using the Boltzmann sigmoidal curve and further derivation to find the transition pH of TPPS at 4.35.

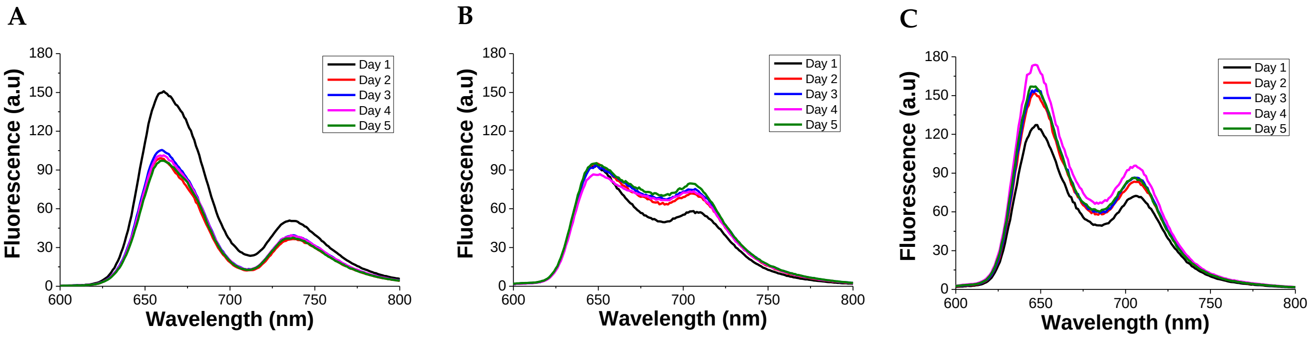

Figure S11. Fluorescence spectra of TPPS at a concentration of 5×10^{-5} M and different pH in the presence of PKHEDA 1×10^{-4} M. pHs: (**A**) 2.92, (**B**) 6.29, (**C**) 10.7. Excitation slit 5 nm and emission slit 10 nm. Excitation wavelength 430 nm.

Reference.

1. Araya-Hermosilla, E.; Roscam Abbing, M.; Catalán-Toledo, J.; Oyarzun-Ampuero, F.; Pucci, A.; Raffa, P.; Picchioni, F.; Moreno-Villoslada, I. Synthesis of tuneable amphiphilic-modified polyketone polymers, their complexes with 5,10,15,20-tetrakis-(4-sulfonatophenyl)porphyrin, and their role in the photooxidation of 1,3,5-triphenylformazan confined in polymeric nanoparticles. *Polymer* **2019**, *167*, 215-223, doi:https://doi.org/10.1016/j.polymer.2019.01.079.
